# Multisite Simultaneous Neural Recording of Motor Pathway in Free-Moving Rats

**DOI:** 10.3390/bios11120503

**Published:** 2021-12-08

**Authors:** Yiran Lang, Rongyu Tang, Yafei Liu, Pengcheng Xi, Honghao Liu, Zhenzhen Quan, Da Song, Xiaodong Lv, Qiang Huang, Jiping He

**Affiliations:** 1 Beijing Innovation Centre for Intelligent Robots and Systems, Beijing Institute of Technology, Beijing 100081, China; yiran.lang@bit.edu.cn (Y.L.); tangrongyu@semi.ac.cn (R.T.); xiaodong.lv@bit.edu.cn (X.L.); qhuang@bit.edu.cn (Q.H.); 2 School of Mechatronical Engineering, Beijing Institute of Technology, Beijing 100081, China; yafei.liu@bit.edu.cn (Y.L.); 3120185110@bit.edu.cn (P.X.); sdfclhh@163.com (H.L.); 3 Key Laboratory of Molecular Medicine and Biotherapy, School of Life Science, Beijing Institute of Technology, Beijing 100081, China; qzzbit2015@bit.edu.cn (Z.Q.); songda18810276950@163.com (D.S.)

**Keywords:** invasive, motor pathway, electrophysiology, microelectrode array, wireless, spinal cord, peripheral nerve

## Abstract

Neural interfaces typically focus on one or two sites in the motoneuron system simultaneously due to the limitation of the recording technique, which restricts the scope of observation and discovery of this system. Herein, we built a system with various electrodes capable of recording a large spectrum of electrophysiological signals from the cortex, spinal cord, peripheral nerves, and muscles of freely moving animals. The system integrates adjustable microarrays, floating microarrays, and microwires to a commercial connector and cuff electrode on a wireless transmitter. To illustrate the versatility of the system, we investigated its performance for the behavior of rodents during tethered treadmill walking, untethered wheel running, and open field exploration. The results indicate that the system is stable and applicable for multiple behavior conditions and can provide data to support previously inaccessible research of neural injury, rehabilitation, brain-inspired computing, and fundamental neuroscience.

## 1. Introduction

The descending pathway of movement in vertebrates mainly includes motor cortex, spinal cord, peripheral nerve connection with muscle and corresponding muscle tissue. Acquisition of the electrophysiological information of these key tissues provides important data [1] for the research of neural pathways, neurodegenerative diseases [2,3,4], motor function injury and rehabilitation [5,6,7], brain–computer interface [8,9,10,11], etc. At present, numerous motor pathway-related studies focus on one or two adjacent sites [12,13,14,15,16,17]. It is a challenge to interface with large-scale cortical neurons [18]. This limits the synchronous observation and research of motion control pathways to a great extent. If some technology can synchronously acquire and observe multisite information, it will greatly expand our knowledge of motor control pathways.

Cortical neuron recording has been extensively applied in neuroscience research [19,20]. Various electrodes have also been developed, such as the most commonly used microwire electrode [21], silicon probe [22], floating or adjustable microelectrode array [23,24,25], electrode combined with optogenetic fibers [26,27,28,29], and EEG electrode [30,31]. Among them, the electrode array with an adjustable shaft can continuously adjust the depth of the tip of the electrode and obtain a good-quality signal for a long time. It is an ideal cortical acquisition electrode for consciously free-moving animals.

Electrophysiological recording of the spinal cord in animal behavior faces more challenges than that of the cortex given the flexible structure of the vertebrae and the softness of the neural tissue. Patch clamp is the most common approach to acquire single neuron signals of spinal cord in vivo [32,33,34]. However, the number of channels in this technology is unlikely to be enlarged, and it is difficult to apply it to awake animals. Microwires are another option that could be inserted to record extracellular single-unit signals of spinal cord [35,36,37,38,39]. For awake and free-moving animals, a recording probe can be mounted outside the local vertebra [40]. Nonetheless, the volume of the implant outside the spine may influence the behavior of rats. Silicone-based compliant electrodes have also been developed for interfacing with flexible tissues. For instance, Stephanie Lacour at EPFL invented an e-Dura implant that is capable of long-term recording, stimulating, and drug delivery of spinal cord [41,42]. Regardless of which technique is applied, it is optimal to connect the electrode or electrode array to the connector and fix it on the skull [43], if it is not wireless.

As the last chain of the nervous system in the motor control pathway, the peripheral nerve plays an important role in signal transmission. Its signals directly innervate muscles to produce contraction and movement. In order to decode motor commands from peripheral nerves, many studies developed various electrodes to interface with peripheral nerves (for review, see [44,45,46]), including the cuff electrode, the flat interface nerve electrode (FINE) [47], the longitudinal intrafascicular electrode (LIFE) [48], etc. For instance, a microchannel scaffold was fabricated to interface with a sciatic nerve amputee for regeneration [49]. Researchers implanted EMG electrodes and a microchannel neural interface simultaneously to monitor sciatic nerve regeneration and muscular signal in awake animals [50]. Another study fabricated a multichannel electrode with silicone rubber and elastic thin film that recorded motion-related signals for over ten weeks [51]. A helix-shaped memory electrode was invented for peripheral nerve stimulation and recording [52]. A wireless interface unit was also developed and utilized in rodents, which could be used in future clinical applications [45].

In consideration of the limitation in current in vivo multisite recording, especially for the whole motor pathway of freely moving animals, we propose a solution of multisite recording of neural and muscular systems in rats in conjunction with kinematics using various electrodes and transmitters, including an adjustable microwire electrode for cortical neurons, a floating multichannel electrode array for spinal cord, a wireless transmitter together with a cuff electrode for peripheral nerves, and bipolar microwire electrodes for EMGs. This technology acquires multisite information in the motor pathway in free-moving rats simultaneously, which provides important information for motor system injury, rehabilitation, mechanical intelligent control, brain-inspired computing, and fundamental neuroscience.

## 2. Materials and Methods

### 2.1. Animals

Four SD rats (weighing 300 ± 50 g) were used in the study. The animals were raised in a sound-insulated cage with corncob bedding for one week to adapt to the environment. Food and water were supplied ad libitum. The temperature was set at 25 ± 2 °C, with natural light circulation. Thereafter, the animals were trained on a treadmill and wheel every day. The study was approved by the ethics committee of Beijing University of Technology.

### 2.2. Design and Function of the Transmitter Module

**Figure 1:**
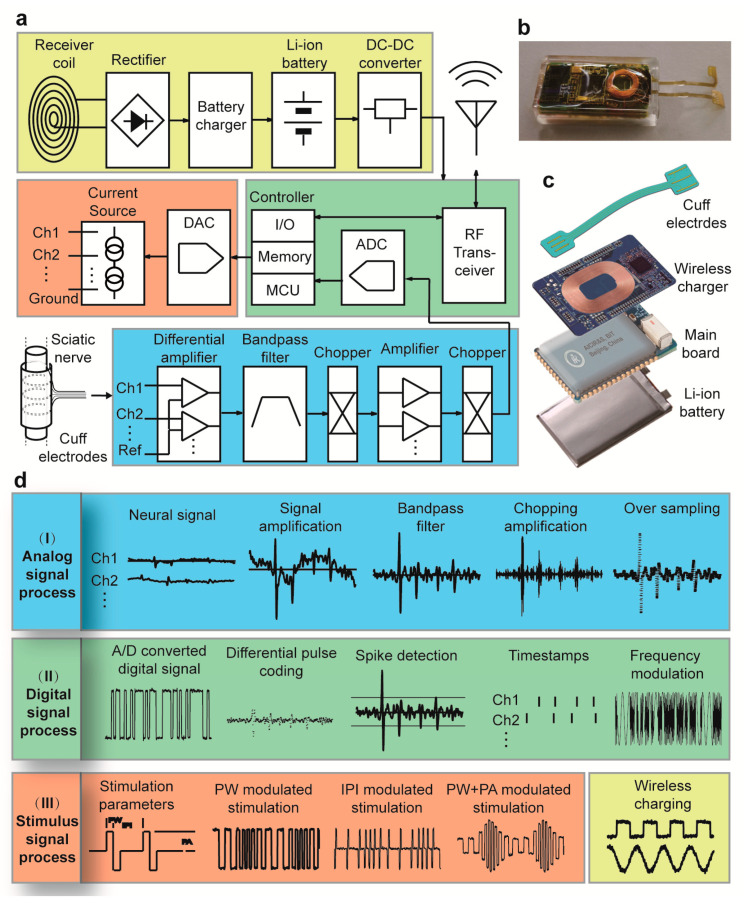
The system block diagram, photograph, 3D assembly, and signal processes of the implantable wireless neural interface microsystem (Neurobits). (**a**) The system block diagram of Neurobits, which consists of an analog signal acquisition unit, a digital signal process unit, a stimulation unit, and a wireless charger. (**b**) Photo of Neurobits. (**c**) Three-dimensional view of the system assembly, consisting of a cuff electrodes array, a wireless charger board, a main board, and a Li-ion battery. (**d**) Diagram showing how different signals are processed inside Neurobits (I, II, III). (I) In analog signal processes, the neural signal from electrodes is amplified and band-pass filtered before passing through a chopper amplifier to reduce internal offset voltage and low-frequency noise. Finally, the analog signal is oversampled to decrease noise and converted into a digital version. (II) In digital signal processes, the signal is encoded using the differential pulse coding method to reduce bit flow. Neurobits is also capable of detecting neural spikes and recording their timestamps. The coded raw signal or spike timestamps are wirelessly sent out through the RF transceiver using the frequency modulation method. (III) In stimulus signal processes, stimulation parameters are remotely set and sent wirelessly to Neurobits, which generates three different types of stimuli according to these parameters, i.e., pulse width-modulated stimuli (PWMS), interpulse interval-modulated stimuli (IPIMS), and pulse width plus pulse amplitude-modulated stimuli (PW+PAMS).

An implantable wireless neural interface microsystem (Neurobits) was custom-made to record the sciatic nerve signal (Figure 1a,b). A 3-channel thin-film cuff electrode is integrated with Neurobits. The electrodes array was fabricated on a flexible polyimide substrate and comprised three evenly spaced gold electrodes with dimensions of 2.5 × 0.3 mm^2^ and a gap of 1.5 mm between them, two of which were connected to the signal differential inputs and one to the signal reference. A 15 µm thick polyimide-based copper-clad film (Upisel, UBE Ltd., Tokyo, Japan) was sputter coated with a layer of Au (150 nm). The Cu/Au layer was then photolithographically patterned and wet etched to give the shape of the electrodes array. A 10 µm thick layer of polyimide (PI-4520, HD MicroSystems Inc., Tokyo, Japan) was spin-coated, photolithographically patterned, and thermally polymerized to form the insulation layer. The electrodes array was soldered to the circuit mainboard of Neurobits (Figure 1c). The sciatic nerve signal collected by cuff electrodes was first amplified in the signal acquisition unit by 500 times. The signal was also band-pass filtered to remove DC voltage offsets and high-frequency noise (1 Hz–5 kHz) before passing through a chopper amplifier to reduce internal offset voltage and low-frequency noise. In order to reduce noise, the neural signal was oversampled at 10 kHz. The oversampled signal was then downsampled with the average method and digitized at a final frequency of 2 kHz before sending to the wireless data transmission unit (Figure 1d). To achieve both insulation and biocompatibility, Neurobits was double encapsulated in a first layer of epoxy resin (LOCTITE^®^ M-21HP, Henkel Corp., Rocky Hill, CT, USA) and a second layer of medical adhesive silicone (SILASTIC^®^ Type A, Dow Corning, Midland, MI, USA). The system is wireless switchable and rechargeable after implantation. The integrated Li-ion battery supports a 4-h continuous working period. The system was tested to be valid after a month of implantation in rat.

### 2.3. Implantation of Electrodes

#### 2.3.1. Motor Cortex Electrodes

**Figure 2:**
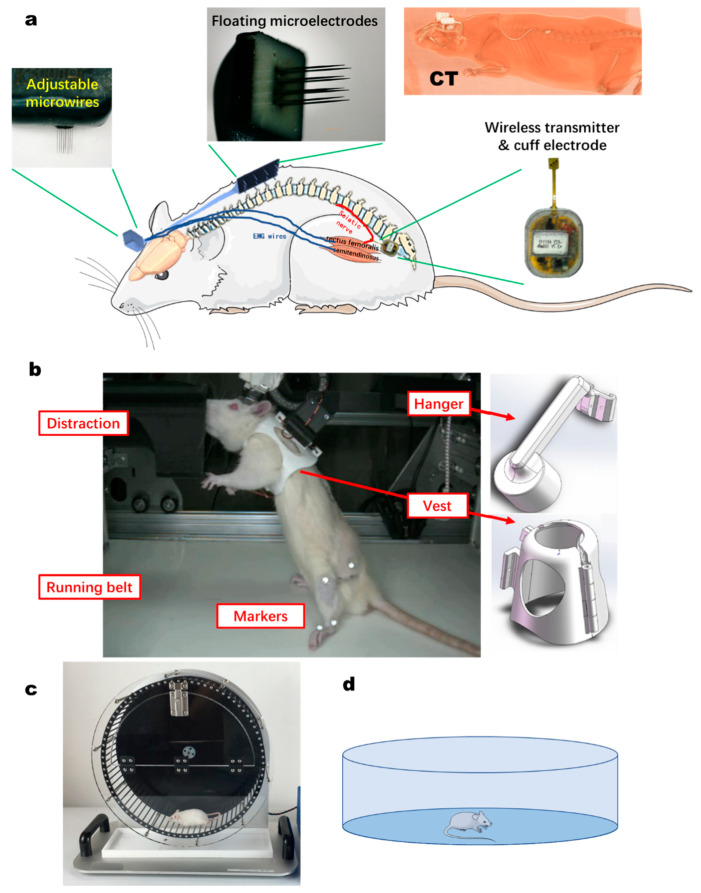
Positioning of implanted electrodes and experimental setups. (**a**) Schematic diagram of implanted electrode and wireless transmitter position. The motor cortex recording electrode adopted an adjustable microwire electrode (2 × 8-channel). Spinal cord signals were recorded by a floating multichannel electrode array (fMEA, 2 × 4 channels). Paired coated microwires recorded EMG signals inserted into the rectus femoris and semitendinosus muscles. The sciatic nerve signals were collected by a self-developed wireless transmitter. Upper right: CT imaging of position of electrodes. (**b**) Home-made treadmill. The rat treadmill system consists of a treadmill belt, suspension system, and control system. A cylinder was placed in front of the animal to distract the animal. With pasted markers, the joints’ coordinates were recorded by high-speed videos. (**c**) Running wheel. (**d**) Open field chamber.

The motor cortex recording electrode uses an adjustable depth 2 × 8-channel microwire electrode (MWE, Kedou (Suzhou) brain computer technology Co., Ltd., Suzhou, China). The average impedance of nitinol microfilaments measured at 1000 Hz and 0.1 V was 1.5 MOhm. The microfilament space was >200 µm and the adjustable length was 4–7 mm. The head was fixed on the stereotactic instrument, and the skin and membrane tissue of the rats were incised from the midline. The skull was exposed, the bregma was considered as the origin, and the right motor brain area was identified (AP: 1.25–3.75 mm; ML: 2.5 mm; DV: 1.5–2.0 mm). The skull was drilled with a skull drill and the dura mater was carefully removed. After implantation, the electrode and screw were fixed together with dental cement (Figure 2a).

#### 2.3.2. Spinal Cord Recording

Spinal nerve signals were recorded by a floating multichannel electrode array (fMEA, 2 × 4-channel, Kedou (Suzhou) Brain-computer Technology Co., Ltd., Suzhou, China). The tungsten needle electrode has an impedance of around 8 KOhm and a length of 4 mm. The L1-L2 segments of the vertebrae were located through the sternum and spinal process. The skin was opened over the position, the muscle tissue and membrane tissue attached to the outside of the spine were peeled, and the vertebrae were exposed. A 2 × 3 mm hole was made with a cranial drill, avoiding damage to the spinal cord. The fMEA electrode plate was fixed in the bone window with a small amount of dental cement. The muscles were sutured with absorbable sutures. Then, fMEA wire was subcutaneously embedded and the skin was sutured with non-absorbable suture. The interface connector was fixed on the head together with cortex MWE (Figure 2a).

#### 2.3.3. Sciatic Nerve Interface

The left hindlimb was shaved and disinfected. Incisions (3 cm long) were made in the hindlimb below the femur. Superficial muscles covering the nerve were separated and the sciatic nerve was exposed. The distal end of the thin-film cuff electrodes was inserted, folded around the sciatic nerve, and closed by suturing, approximately 15 mm proximal to the point where it branched into the tibial and peroneal nerves. Neurobits was placed under the skin and tested with a trial signal recording before the incision was closed by suturing (Figure 2a).

#### 2.3.4. EMG Microwires

Paired coated microwires (50 microns core) were embedded in the agonist and antagonist muscles to record the electromyography of a muscle. First, the signals collected by pairs of microwires were differentiated to obtain a relatively clean EMG signal. Microwires were subcutaneously passed through and connected on the skull (Figure 2a).

### 2.4. Recording during Tasks

#### 2.4.1. Bipedal Treadmill Locomotion

Each rat was suspended on a homemade treadmill (Figure 2b) and bipedally walked on hindlimb. The behavior research system consisted of a rat treadmill, a suspension system, a computer, and accessories. The hanging vest of each rat was three-dimensionally printed according to the different animal sizes. The suspension angle of the upper body could be fixed or adjusted to keep the lower limbs walking upright, while avoiding interference of the upper limbs. A cylinder was placed in front of the animal face to appease the animal and provide a bar for the upper limb to grasp. The speed range was 0.015–0.035 m/s and was determined according to the animals’ adaptation. Before electrode implantation surgery, the animals were trained on the treadmill for 10–30 min every day until they were capable of steady walking for more than 10 min.

The motor cortex and spinal cord signals were recorded using the Plexon system (Plexon Inc., Dallas, TX, USA), with a sampling rate of 40 kHz. The collected raw signals (wide band) of the cortex and spinal cord were preprocessed using an offline sorter (v4.3.1, Plexon Inc., Dallas, TX, USA). First, the Bessel low-pass filter removed the low-frequency part of the signal (<350 Hz). Thereafter, 3.5 times the standard deviation was used to automatically develop a threshold for the signals of all channels. The t-dist E-M method (d.o.f multiplier was 10; outlier threshold was 2) was utilized for signal sorting. The raw signal, low-frequency signal, continuous high-frequency signal, and spikes were saved in separate files. Four industrial cameras were used to conduct video recording synchronously, with a frame rate of 80 FPS and a resolution of 640 × 480. In order to obtain a better tracking effect, four 5-millimeter reflective markers were respectively pasted at the hip joint, knee (lateral condyle), ankle (lateral malleolus), and mtp (fifth metatarsophalangeal). The two-dimensional coordinates of the markers were obtained using Simi Motion2D (SIMI Motion version 9.2.2, Simi Reality Motion Systems GmbH, Unterschleissheim, Munich, Germany) to represent the spatial position changes of each joint.

The implantable wireless neural interface microsystem (Neurobits) and the integrated thin-film cuff electrodes were custom-made to record the sciatic nerve signal. Microwires were inserted to record the rectus femoralis and semitendinosus muscles, which are responsible for extension and flexion of the knee joint.

#### 2.4.2. Wheel Running

The inner diameter of the running wheel (Originopto Ltd., Hangzhou, China) was 340 × 100 mm, the distance between wheel rods (center distance) was 13.5 mm, and the speed range was 0.1–20.0 turns/min (Figure 2c). Before the electrodes were implanted, the animals underwent 10–30 min of running wheel training every day until the animals could walk stably in the wheel. The recording methods of motor cortex, spinal cord, sciatic nerve, and motor information were the same as those described above.

#### 2.4.3. Open Field Walking

The diameter of the open field chamber was 95 cm (Figure 2d). The recording methods of motor cortex, spinal cord, sciatic nerve, and motor information were the same as those described above. In order to avoid entanglement of the two electrode cables during free movement, a commutator (Plexon Inc.) was used to untwist the two electrodes. The camera only recorded the central position of the animal trunk, not the position of the joints. The equipment was cleaned with alcohol after each experiment to avoid leaving odor traces for the next animal.

## 3. Results

### 3.1. Multisite Simultaneous Recording in Motor Pathway

**Figure 3:**
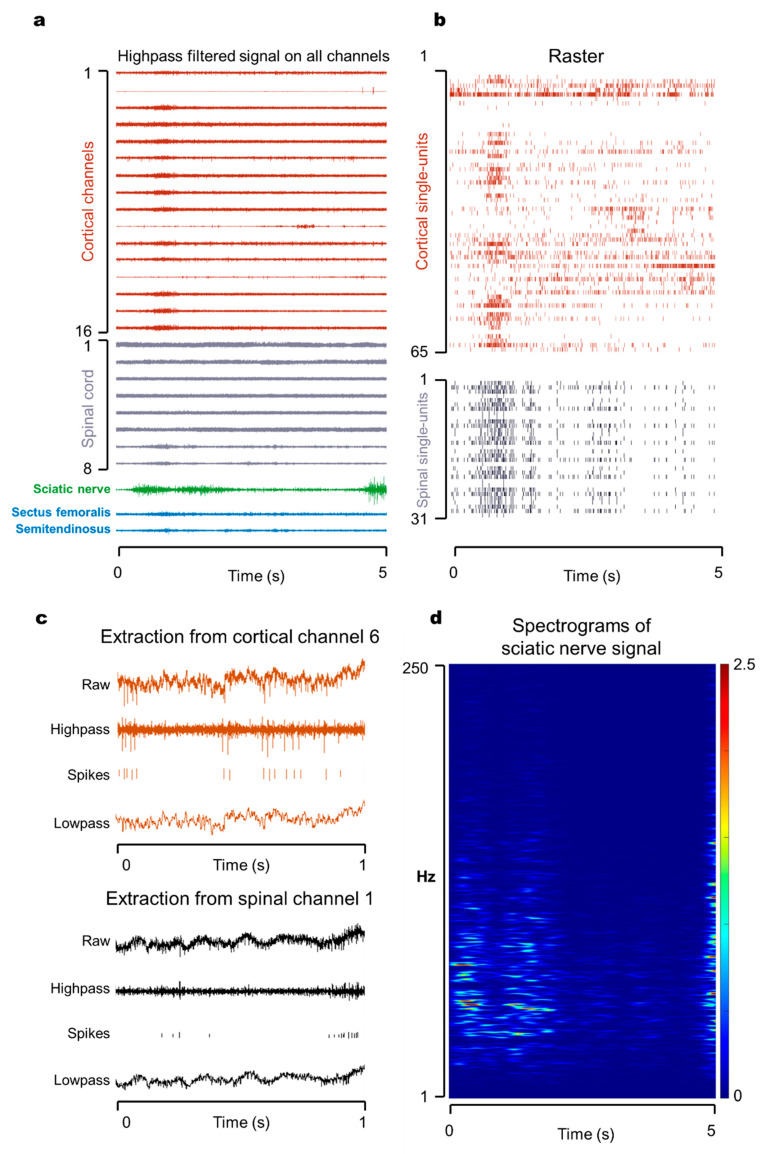
Electrophysiological data from freely moving rodents. Demonstration of wide-band (1–4 kHz) recording of neural and muscular data using wired and wireless sensors from which more information can be further extracted. (**a**) Graph of multisite simultaneous neural recording of motor pathway over a 5-s duration. Cortical channels in red; spinal cord channels in grey; sciatic nerve signal in green; EMGs in light blue. (**b**) Plot of raster of single unit of cortical and spinal cord data over the same time duration. (**c**) Example of information extraction on a single channel (channel 6 for cortical signal and channel 1 for spinal cord signal) over a 1-s time window. High pass and low pass was divided from 350 Hz. Waveforms larger than 3.5 × standard deviation were isolated as spikes. (**d**) Spectrograms of sciatic nerve signal ranging from 1 to 250 Hz over the same 5 s.

We have developed technology to enable simultaneous in vivo recording of signals of motor cortex neurons, spinal nerves, peripheral nerves, and muscles (Figure 3a). Meanwhile, the joint 2D coordinate information was collected and reconstructed using a high-speed camera and motion capture software. In this way, the electrophysiological information of the whole motor pathway was acquired. The cortical, spinal cord, and muscle signal electrodes were subcutaneously connected to the connectors fixed on the skull and interfaced with the acquisition system. The self-developed wireless transmitter and a flexible cuff electrode recorded and wirelessly transmitted peripheral neural signals. Synchronization between the wireless and wired systems was achieved by starting the recording at the same time and using an inner clock.

The original signals acquired by this technology were continuous broadband signals. The sampling frequency of cortex, spinal cord, and electromyography was 40 kHz, and that of sciatic nerve was 2 kHz. A continuous broadband signal could be high-pass filtered to retain high-frequency baseline and spikes. The average noise was 33.2 ± 7.4 µV (mean ± SD), and the signal-to-noise ratio was 5.4 ± 3.1. Since the action potential of the cerebral cortex obeys the “all-or-none” principle, spikes were further abstracted as timestamps—that is, a raster diagram (Figure 3b). The low-frequency field potential was retained by low-pass filtering (Figure 3c). The field potential signal was further decomposed into a spectrogram (Figure 3d) to observe the energy change of each frequency band. Both high- and low-frequency signals contain useful motor-related information that can be decoded for the brain–computer interface [53].

The challenge of whole motor pathway recording is the multimodal signal monitoring of animals in the state of awakeness and free movement, especially the recording of spinal cord nerves. Our laboratory implanted an fMEA and fixed it in the spinal cord through a tiny opening on the back of the vertebrae, which could achieve stable recording of spinal cord signals. High integration and miniaturization of the electrodes are the key to this technology. For instance, the diameter of the cable between the fMEA and the connector was 0.8 mm, and the baseplate of probes was 2.18 × 2.26 mm (width × length). The diameter of the coated EMG microwire was 120 µm. The thickness of the cuff was 180 µm, and its width was 4.0 mm for three channels.

### 3.2. Monitoring of Neural and Muscular Activity during Bipedal Walking on a Treadmill

**Figure 4:**
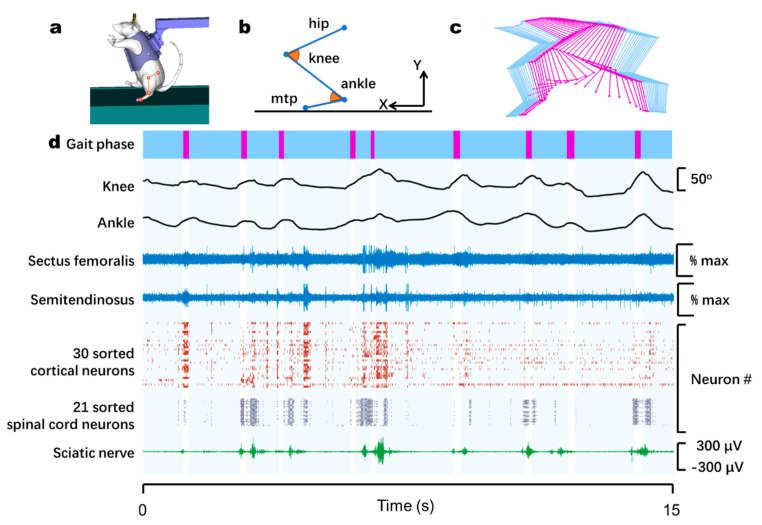
Application of multisite recording for the study of whole motor-related neural pathway and locomotion of bodyweight-supported bipedal walking on a lab-made treadmill. (**a**) An upper-body-fixed rat walking on the treadmill, with an intra-cortical MEA in the M1 leg area, an fMEA in the spinal cord, a wireless transmitter on the sciatic nerve, and EMG recordings from two muscles. The red dots and lines highlight the position of markers magnified in (**b**). The positive X is the stepping direction. The angle of the inner corner of knee and ankle are in orange. (**c**) A skeletal reconstruction of the markers during one cycle of stepping. (**d**) Demonstration of data from one rat. From top: gait phase (purple, swing phase; blue, stance), angle of joints, EMGs of two muscles, raster plot of cortical neurons, spinal cord signals, and continuous potential of sciatic nerve.

First, we tested the performance of our technology for upper-body-restrained bipedal walking on a treadmill (Figure 4a–c). At present, most in vivo electrophysiological techniques only implant electrodes in a single site. This limits the observation of the multimodal information of motion-related pathways at the same time. Our technology can simultaneously record multisite neural and EMG activities when animals are awake and freely moving. We implanted a 16-channel electrode MEA in the leg area of the primary motor cortex, an 8-channel fMEA in the left spinal cord L2, a wireless transmitter on the left sciatic nerve, and paired microwires in two muscles. EMG activity and whole pathway neural data were simultaneously recorded (Figure 4d) with kinematics while rats were walking on a homemade treadmill.

All four rats displayed reproducible modulation of motor cortex and spinal cord spiking activity that appeared across the entire walking period. In addition, burst activity of sciatic nerve were repeatedly appears in each gait cycle. Our multisite recording system provided opportunities for observing the activity of neural populations and muscles in the motor-related pathway during natural bipedal walking.

### 3.3. Recording of Neural and Muscular Activity during Quadrupedal Wheel Running

We next demonstrated the capacity of our system in a free moving situation for research on rodents. The task of wheel running involves untethered but forced quadrupedal walking in a relatively limited space. The speed and direction of movement were controlled by the wheel manipulator. Compared to the treadmill movement under tethered condition, the whole body of the animals quadrupedally moved back and forth in the wheel. The position and angle of the animal trunk varied continuously. If the moving speed of the animal was slower than the speed of the wheel, the animal moved backward and the body posture entered a downhill state with the head down. If the crawling speed was faster than the wheel, it appeared uphill.

**Figure 5:**
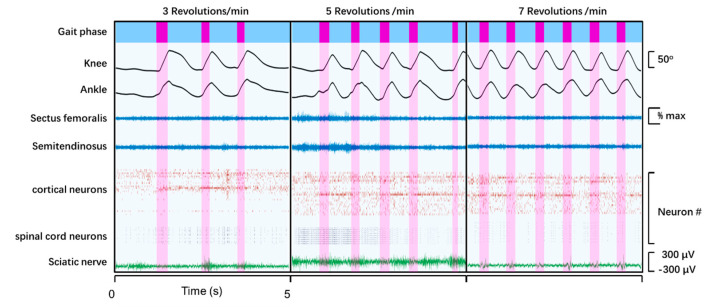
Application of multisite recording for the study of neural network and locomotion kinematics during forced movement in running wheel. Data from the multisite recording system for an animal (rat #4) walking in the running wheel at three different speeds: 3, 5, and 7 revolutions/min (rpm). From top: gait phase, joint angle plots at the knee and ankle, EMG signals from two muscles, raster plots of sorted cortical and spinal units, and the sciatic nerve signal during walking.

The temporal repeated pattern of cortical and spinal cord neuronal ensemble covaried with the revolution speed changes (3, 5, and 7 rpm) in joint angles and leg muscles (Figure 5). The results showed that our recording system had three-dimensional spatial flexibility and a stable connection during the whole movement. In addition, the wireless transmitter continuously received sciatic nerve signals, without interference or noise from the plexiglass partition and the wheel control system.

### 3.4. Investigation of Signal Transitions in Open Field Conditions

**Figure 6:**
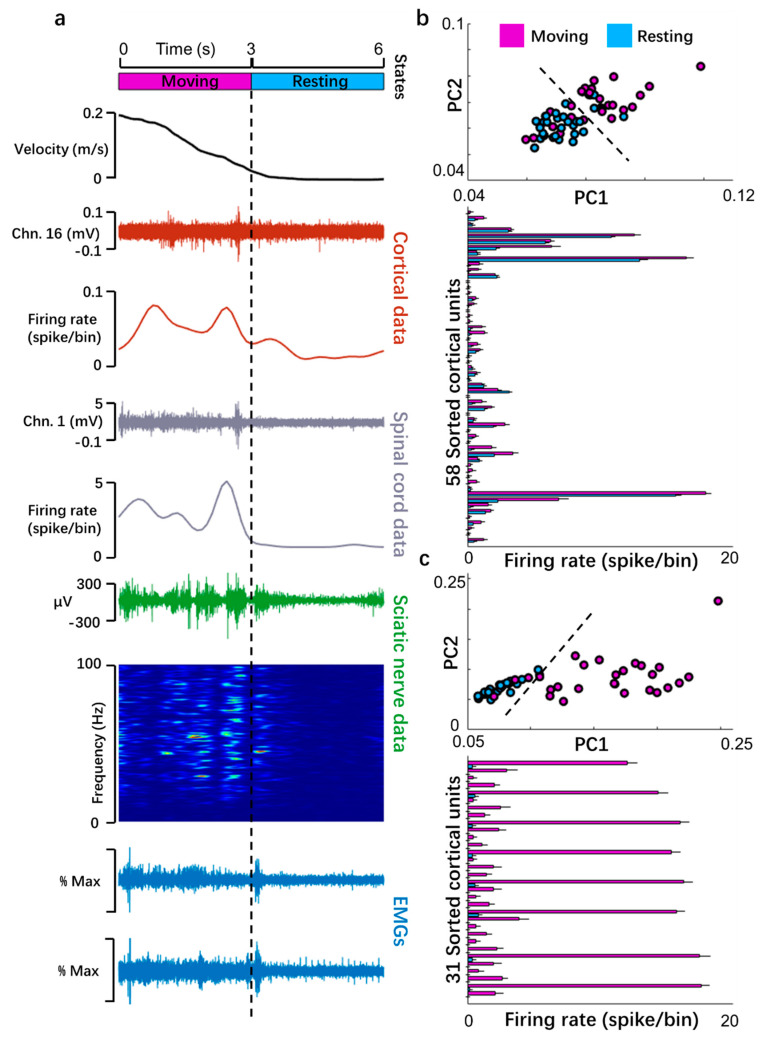
Sample data of long-term recording from a rat in an open field. (**a**) Sample data from 6-second moving-to-rest transition. From top: velocity of trunk movement; continuous signal and firing rate of a channel in motor cortex in spike/bin, where bin = 0.1 s; continuous signal and firing rate of a spinal cord channel; continuous signal and spectrograms of sciatic nerve; EMGs of rectus femoralis and semitendinosus muscles. (**b**) PCs and firing rate of cortical neuronal assemblies. Top: 2D PCA space of the 3 s data of the two states clearly indicating that it can be divided into two groups relying on principal components of cortical signals. Bottom: Comparison of firing rate of well-sorted units. (**c**) PCs and firing rate of spinal cord channels.

Next, we tested the system in a larger space (circular plane with a diameter of 85 cm). The open field task prompts two requirements for the signal acquisition technology. First, the wired segment allows the animals move freely in the field without interference to activities, and the wireless part should be able to receive signals reliably. Second, multichannel data acquisition can be sustained for a relatively long time. We video-captured the activities of four rats in the chamber for 10–20 min and continuously recorded all the signals. This broadband recording technique helped to distinguish the electrophysiological signals between moving and resting states. The explicit transformation of these signals is illustrated in Figure 6a.

The activities of neuronal assemblies during the behavioral transition from a moving to a resting state were preliminarily studied. The results showed that 81.03% (47 out of 58 sorted units, Figure 6b) of cortical neurons were significantly inhibited (*p* < 0.05). We then applied a PCA on the firing rates for all the recorded units. Two separate clusters dependent on the first two principal components illustrated contrasting neuronal population dynamics during the moving and resting states. Similarly, PCs distinguished spinal cord neurons into two clusters affiliated to the two states. Furthermore, the firing rate of the spinal cord decreased more dramatically during the transition than that of cortical neurons for 100% (*p* < 0.05, 32 out of 32 units, Figure 6c).

We found that endpoints differed between trunk ceasing forage and electrophysiological signals decrease. It is possible that the rats balanced their bodies in the resting state by nuanced angle adjustment of limb joints, which was difficult to detect by motion capture in video but easily observed in signals of sciatic nerve and EMGs. During natural exploration behavior, we found shifts in power between 20- and 100-hertz frequency bands in the spectrum of sciatic nerve LFPs, which corresponded to periods of marked moving and resting states relying on movement velocity. This indicated that the hindlimb-related information was mined in the gamma band, but not in the low-frequency band (Figure 6a).

Therefore, this technology provided the opportunity to observe multipoint and multichannel electrophysiological information during long-term and large-scale activities of animals.

## 4. Discussion

In this study, we built a system that integrates electrodes targeting the cortex, spinal cord, peripheral nerves, and muscles and an acquisition system for the broadband electrophysiological signals of these tissues. Through the treadmill, wheel, and open field tests, our system was proven to adapt to the different requirements of partially tethered, free moving in limited space, and large open space experiments. The system stably recorded multisite electrophysiological signals of awake and freely moving animals for a long time, which were previously inaccessible. We also evaluated the different components of these signals, such as LFP, low-frequency band, high-frequency band, and spikes. These data provide an opportunity for various studies to monitor the motor neural pathway in natural states.

Electrophysiological techniques are often used in many fields of research, such as neural circuits, nerve injury, and basic neuroscience. For example, there are studies on the simultaneous implantation of two [54] or three electrodes in different cortical motor areas [55]. Some studies have recorded multiple neuronal signals in the motor cortex and EMG signals of contralateral leg to study spinal cord injury [56]. Others have used different electrodes to simultaneously record neuronal signals in the cerebral cortex and spinal cord [41]. These studies addressed the value of multisite recording of motor-related pathways. Compared with other studies, the present technology provides more comprehensive information.

The spinal cord is a vital segment between the central nervous system and the peripheral nervous system. Most of the downward motor commands and upward sensory information are transmitted through the spinal cord. However, due to the limits of appropriate acquisition and recording technology, tissue slice staining and reversible or irreversible damage approaches were commonly used for the study of spinal cord. Our data of cortex and spinal cord imply that although the spike signals of both tissues are related to the motion process, the units in the cortex are more diversified, suggesting that neurons in the motor cortex have more functional diversity than the spinal cord. In contrast, the fluctuation of spinal cord nerve discharge is highly correlated with lower limb movement, which indicates that the function of the spinal cord is more specifically related to lower limb movement. In terms of noise, the cortical and spinal cord signals render a fine SNR to extract a clear waveform distinguished from noise after manually invalidating artifacts. The noise level and SNR are not better than those of high-density silicon probes [22] but are comparable with electrodeposited platinum–iridium-coated electrodes [57].

The sciatic nerve is a major player in the nervous system to innervate muscles and produce contraction and extension. It assembles a bundle of nerve fibers. The signals acquired by cuff are usually much smaller than the potential transmitted along the peripheral nerve. Therefore, the data recorded by the cuff electrode are mainly displayed as field potentials. The most obvious signature of hindlimb motion was found to be concentrated in the gamma band of 20–100 Hz. In summary, different tissues in the motor pathway have different electrophysiological activities, suggesting that different analysis methods are required for different signals in future research. In order to extract spikes for decoding, some researchers adopted a wavelet for denoising [58]. One study developed an implant carrying an eight-by-twelve matrix of parallel microchannels. The wavelet denoising method was also utilized to generate clear spikes that are four times the SD of baseline noise [51], which is better than our raw sciatic signals. It suggests that the quality of the sciatic nerve of the present study could be further enhanced using a denoising algorithm for the decoding study.

Notably, the data from the wheel task were different to those from the treadmill task. This may be because the animals needed to walk with their hind limbs on the treadmill and maintain a rear position of the body, so the multimodal nerve signals showed a temporal repeated pattern [59]. On the wheel, the rats walked with all four limbs, so the pressure on each limb was relatively dispersed, which invokes less strength in the downhill phase [30], so the fluctuation of signals was relatively weak. In the open field task, the neural activities of the motor cortex and spinal cord ceased while the trunk movements stopped, but the sciatic nerve activities and EMG signals remained active. This may be because the animals subconsciously adjusted the posture of the limbs at the end of the movement, but the cerebral cortex was not required to participate in this process.

Numerous studies implanted microwires or an MEA into the cerebral cortex and muscle tissue for a long duration (>6 months) and found no serious inflammation or immune reaction [60,61,62]. We implanted fMEAs into the spinal cord for more than one month at most and the arrays were working. Moreover, no adverse reactions and behavior were found. A self-developed chip and the thin-film cuff electrode, Neurobits, for recording and transmitting sciatic nerve signals were tested after long-term implantation in rats. Implantation had no significant impact on animal behavior. After one month, the system continued to work normally and transmitted nerve signals. In the future, the electrode volume could be further reduced in order to improve the surgical process and minimize the discomfort to the animals.

The cortex, spinal cord, and peripheral nerve together constitute a complete motor nerve pathway. Their signals reflect the motor-related information of the nervous system at different stages and aspects, such as perception, processing, transmission, and execution. There are crucial differences and correlations between these signals. The signals collected by cortical electrodes are the reflection of complex brain activities in motion-related brain areas, including a large amount of comprehensive information such as motion perception, planning, control, etc. The spinal cord is the main transmission channel between the central and peripheral nervous systems and has some primary reflex central functions. Spinal cord signals contain sensory and motor information from the skin, muscles, and throughout the body. Sciatic nerve signals are highly specific, mainly including sensory terminal information of the lower limbs and muscle movement control instructions. Research on movement-related nervous system injury diseases, brain–computer interface, neural prosthesis, etc., needs to obtain as much nervous system information as possible. Observation of the whole neural transmission pathway of the cortex, spinal cord, and peripheral nerve is conducive to locate the functional zoning of the nervous system, interpret the characteristics of signals, reveal the processing mechanism of the nervous system, explore the treatment methods of neurological diseases, and develop more accurate and efficient and less damaged brain–computer interfaces. On the contrary, if we only observe the neural activity of the cerebral motor cortex and omit the observation of spinal cord nerves, it is not possible to understand how the downward motion information is compressed and encoded into muscle units and how the sensory signals are amplified to the whole sensory cortex through the spinal cord.

## 5. Conclusions

In this paper, we introduced a solution that can chronically read out the central and peripheral nervous systems. To the best of our knowledge, this is the first study to capture multisite neural signatures (e.g., spiking data, LFP, etc.) across a population of neurons in the cortex, spinal cord, and sciatic nerve as well as EMG and kinematics while the animal moved freely. The elasticity of the fMEA and cuff electrode met the demand of spinal and peripheral nerve tissues. The combination of various implants and a transmitter enables high-resolution and long-term neuronal and muscular recordings in conjunction with kinematics while animals are freely moving. It can be applied on animals in tethered or untethered conditions for studies of motor system injury, rehabilitation, mechanical intelligent control, brain-inspired computing, and/or fundamental neuroscience.

## Data Availability

The data presented in this study are available on request from the corresponding author. The data are not publicly available due to the need for further analysis.
